# Supplementary material for: Role of Graphene in Constructing Multilayer Plasmonic SERS Substrate with Graphene/AgNPs as Chemical Mechanism—Electromagnetic Mechanism Unit
Source: Nanomaterials (Basel). 2020 Nov 28;10(12):2371. doi: 10.3390/nano10122371 (PMC7760367; doi:10.3390/nano10122371)
Supplement: Supplementary file 1 [file nanomaterials-10-02371-s001.pdf]

nanomaterials

Supplementary Materials

Role of Graphene in Constructing Multilayer Plasmonic SERS Substrate with Graphene/AgNPs as Chemical Mechanism—Electromagnetic Mechanism Unit

Lu Liu [1], Shuting Hou [1], Xiaofei Zhao [1], Chundong Liu [1], Zhen Li [1], Chonghui Li [1,2,3], Shicai Xu [2], Guilin Wang [1], Jing Yu [1], Chao Zhang [1,*] and Baoyuan Man [1,*]

[1] Collaborative Innovation Center of Light Manipulations and Applications, School of Physics and Electronics, Institute of Materials and Clean Energy, Shandong Normal University, Jinan 250358, China; 2018020529@stu.sdnu.edu.cn (L.L.); 201809020414@stu.sdnu.edu.cn (S.H.); 2018010048@stu.sdnu.edu.cn (X.Z.); 2019010052@stu.sdnu.edu.cn (C.L.); lizhen19910528@163.com (Z.L.); 2015020674@stu.sdnu.edu.cn (C.L.); 2019020521@stu.sdnu.edu.cn (G.W.); yujing1608@126.com (J.Y.)

[2] Shandong Key Laboratory of Biophysics, Institute of Biophysics, Dezhou University, Dezhou 253023, China; shicaixu@dzu.edu.cn

[3] Institute for Integrative Nanosciences, IFW Dresden, Helmholtzstraße 20, 01069 Dresden, Germany

* Correspondence: czsdnu@126.com (C.Z.); byman@sdnu.edu.cn (B.M.)

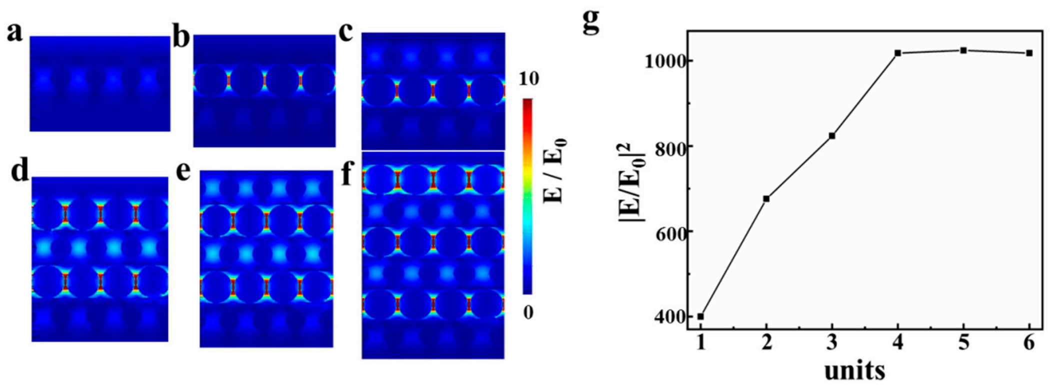

Figure S1. The x-z views of electric field distribution of different number of CM−EM units substrate at 532 nm wavelength: (**a**) one unit, (**b**) two units, (**c**) three units, (**d**) four units, (**e**) five units, (**f**) six units. (**g**) Electric field enhancement ($|E/E_0|^2$) for substrate with different CM−EM units.

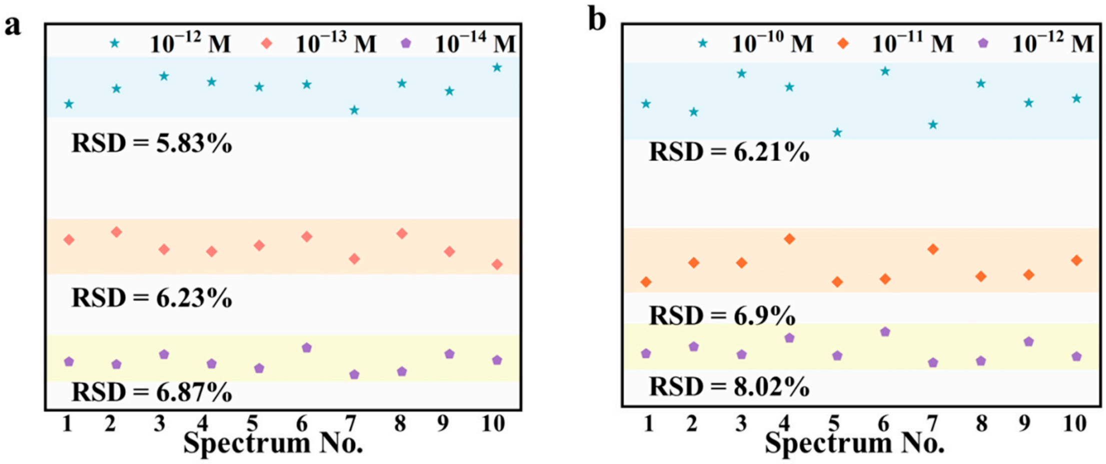

.

Figure S2. The Raman signal intensity distribution of (**a**) R6G at 613 cm^{-1} and (**b**) CV at 914 cm^{-1} on multilayer substrate with four CM-EM unit respectively from 10^{-12} M to 10^{-14} M and from 10^{-10} M to 10^{-12} M.

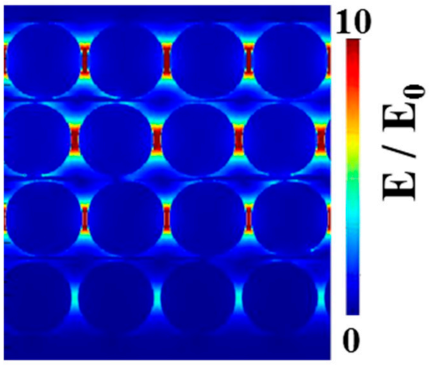

Figure S3. The x–z views of electric field distribution of substrate shifted horizontally.
